# Supplementary Light Source Affects Growth, Metabolism, and Physiology of* Adenophora triphylla* (Thunb.) A.DC. Seedlings

**DOI:** 10.1155/2019/6283989

**Published:** 2019-05-07

**Authors:** Ya Liu, Xiuxia Ren, Byoung Ryong Jeong

**Affiliations:** ^1^Division of Applied Life Science (BK21 Plus Program), Graduate School, Gyeongsang National University, Jinju 52828, Republic of Korea; ^2^Institute of Agriculture and Life Science, Gyeongsang National University, Jinju 52828, Republic of Korea; ^3^Research Institute of Life Science, Gyeongsang National University, Jinju 52828, Republic of Korea

## Abstract

*Adenophora triphylla* (Thunb.) A.DC., a well-known herbaceous medicinal species, has been reported to protect against human obesity, cancer, and inflammation. Supplementary lighting is a practical strategy to improve crop quality, especially at a propagation stage. However, there has been no study available on the optimal supplementary light source for the commercial production of* A. triphylla* seedlings. In this study, plug seedlings were cultivated in a greenhouse for four weeks under an average daily light intensity of 490 *μ*mol·m^−2^·s^−1^ PPFD coming from the sun and a supplemental lighting (16 h per day) at 120 *μ*mol·m^−2^·s^−1^ PPFD provided by high pressure sodium (HPS), metal halide (MH), far-red (FR) light, white LED (red: green: blue = 2:4:3, LED-w), or mixed (red: green: blue = 4:1:4) LED (LED-mix). The results showed that LED-mix, with a higher percentage of red and blue light, substantially promoted seedling growth compared to other treatments by increasing stem diameter, biomass, specific leaf weight, and root to shoot ratio. The LED-mix also promoted accumulation of soluble sugar, starch, and chlorophyll in the tissue and increased contents of total phenols and flavonoids. Moreover, stomata density and pore area per leaf area under the LED-mix were remarkably greater than those under other treatments. Furthermore, the Western blot analysis revealed that the expression of photosynthetic protein, D1, was notably enhanced by the LED-mix as compared with other light sources. In addition, the LED-mix alleviated the oxidative damage of seedlings by improving enzymatic and nonenzymatic antioxidant systems. Collectively, these results suggest that the LED-mix was the optimal supplementary light source for the production of highest quality* A. triphylla* seedlings.

## 1. Introduction

*Adenophora triphylla* (Thunb.) A.DC. (Campanulaceae), also named as three-leaf lady bell or Japanese lady bell, is a perennial herb, which is mainly distributed in Korean Peninsula, China, Japan, and Russia (Far East and Eastern Siberia) [1, 2]. Besides its ornamental value,* A. triphylla* has been an important medicinal plant in oriental medicine for remedying whooping cough and chronic bronchitis in China [1]. In addition, this herb has also been used as a food source to prevent obesity in traditional Korean recipes [3]. In recent years, antitumor and antidiabetic activities have been reported in this species [4, 5]. Some previous studies attached the importance to extraction and identification of phytochemicals, such as lupenone, daucosterol, and adenophoric acid methyl ester, and to the action of disease resistance [3–5]. However, few studies have focused on the effect of light on the growth and development of this plant species.

Light is the most important environmental factor affecting photosynthesis and thus yield because plant growth and yield depend on photosynthesis [6, 7]. Plant morphology, physiology, and biochemistry also vary largely with the light source [8–10]. Light spectrum influences photosynthetic process by adjusting stomatal development and movement [11], photosynthetic pigment level [12], and photosynthetic protein biosynthesis and activities of light-harvesting complexes (LHC), photosystem I (PSI), photosystem II (PSII), and cytochrome b6f (Cyt b6f) [13]. Monochromatic light, especially red and blue light, has been widely studied [14, 15] for their effects. For example, many studies proved that red light affects stem elongation, leaf extension, bud outgrowth, and photosynthetic apparatus [11, 16], while blue light influences leaf thickness, hypocotyl elongation, photosynthesis, biosynthesis and accumulation of secondary metabolites, and stomata development and opening [10, 17, 18]. However, monochromatic light is not sufficient for growth and development for some plant species, leading to lower biomass, abnormal leaf morphology, and reduced photosynthesis as compared with mixed light [18–21]. Therefore, broad-band or dichromatic light source may have to be employed as an artificial light source in some horticultural plant species and their beneficial effects and the molecular mechanism involved should be focused on and deeply studied in the future.

Supplementary lighting is an important horticultural practice and strategy to improve crop growth and to obtain all-year-round, high-yield, and superior-quality productions in greenhouses [19, 22]. In commercial productions, plants are usually provided with an additional lighting at an intensity between 100 and 200 *μ*mol·m^−2^·s^−1^ photosynthetic photon flux density (PPFD) for up to 16 h per day (even 20 h in some regions) to maximize production [20, 23]. Source of supplementary light is, therefore, a key factor in determining the effect of this practice. Traditionally, high pressure sodium (HPS) and metal halide (MH) lamps belonging to high intensity discharge (HID) lamps have been the most commonly used artificial light sources for plant research and greenhouse horticulture [18, 24]. Moreover, supplementary far-red (FR) light has been proved to enhance plant biomass, stem length, leaf length, and leaf width [22, 25]. Recently, a new light source, light emitting diodes (LEDs), has gained widespread attention, since it provides a narrow special wavelength band and a high efficiency [12, 26, 27]. However, data are still scarce regarding the choice of light source and how the supplementary light source influences the growth, physiology, and biochemistry of plug seedlings, especially in plug seedlings of medicinal plants.

Different supplementary light sources may influence primary and secondary metabolism in different manners [20, 28]. Previous studies have shown that conventional HID lamps are more efficient in the improvement of metabolites than LEDs [24, 29, 30]. However, there are studies suggesting that LEDs are more advantageous over traditional light sources in increasing primary and secondary metabolites [31–33]. Therefore, more studies are required to provide more evidences to clear the ambiguity, notably in medicinal plants including* A. triphylla*.

In this study, it was hypothesized that mixed LEDs could be an alternative supplementary light source for stimulating growth and metabolism and, therefore, improving quality of* A. triphylla* seedlings as compared with conventional light sources. To test this hypothesis and accelerate the production of high quality* A. triphylla* seedlings for commercial exploitation, HPS, MH, FR, white LEDs, and mixed LEDs were employed as supplementary light sources during a rainy summer season. In the study, spectral characteristics of those light sources, growth characteristics of seedlings grown under those light sources, and accumulation of primary and secondary metabolites in seedlings were investigated. Moreover, to reveal how the light source drives plant growth and affects seeding quality, stomata properties, expression of photosynthetic proteins, and redox homeostasis were also examined. The data obtained may provide a theoretical and practical basis for improving growth and development, and also enhancing the medicinal value of* A. triphylla* seedlings by supplying an optimal light source. The data may also be helpful in guiding the growers for cultivation and management of* A. triphylla* and other medicinal plants.

## 2. Materials and Methods

### 2.1. Supplementary Light Sources and Cultivation Conditions

**Figure 1:**
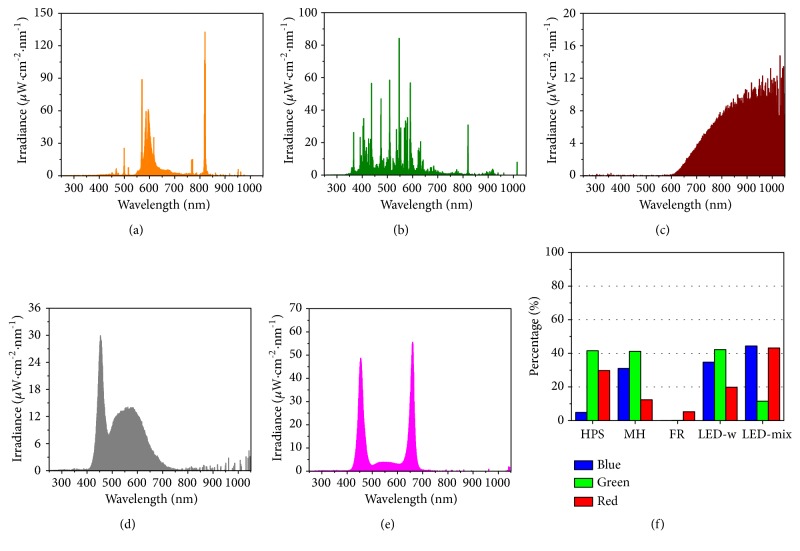
The spectral distribution of high pressure sodium (HPS, (a)), metal halide (MH, (b)), far-red (FR, (c)), white (red: green: blue = 2:4:3) light-emitting diodes (LED-w, (d)), and mixed (red: green: blue = 4:1:4) light-emitting diodes (LED-mix, (e)), and percentages of blue, green, and red (f) light for each supplementary light source used in the study. Irradiance was measured by a traceable calibrated spectroradiometer (ILT950 NIST, International Light Technologies, Inc., Peabody, MA, USA) without sunlight.

In this study, five different light sources (Figure 1), high pressure sodium (HPS, BLV Licht-Und Vakuumtechnik, Steinhöring, Germany), metal halide (MH, SunLumen Lighting Co. Ltd, Gyeongju, South Korea), far-red (FR, Philips Lighting, Amsterdam, The Netherlands), white (red: green: blue = 2:4:3) LED (LED-w, Victory Lighting Ltd, Seoul, South Korea), and mixed (red: green: blue = 4:1:4) LED (LED-mix, Custom made, SungKwang LED Co. Ltd, Incheon, South Korea), were tested. The supplementary light intensity was set uniformly at 120 *μ*mol·m^−2^·s^−1^ PPFD with a 16 h photoperiod, while an average daily maximum light intensity coming from the sun was about 490 *μ*mol·m^−2^·s^−1^ PPFD with a natural photoperiod of 14 h. Seedlings were cultivated in a glasshouse at an average day/night temperatures of 31.6°C/26.3°C (average daily maximum temperature is 34.5°C) and 88.1% relative humidity for four weeks. After cultivation, some plants were harvested for measurements of growth and physiological parameters, and others were frozen in liquid nitrogen immediately after harvest, and then were stored in a −80°C freezer for further analyses.

### 2.2. Analysis of Stomata Using Scanning Electron Microscopic (SEM)

Sample preparation and SEM analysis of stomata were carried out following previously published method [28, 34]. In brief, excised leaves were fixed immediately in a 2.5% glutaraldehyde solution at 4°C overnight. Then samples were stained with 1% osmic acid (OsO_4_) at 4°C for 2 hours. Subsequently, they were dehydrated by a graded series of ethanol, followed by wash with 80% acetone. After fixation and staining, samples were washed carefully by a 0.1 M phosphate-buffered saline solution (PBS, pH 7.0). Finally, dried samples were gold coated, observed, and photographed by using a scanning electron microscope (JSM-6380, Jeol Ltd., Tokyo, Japan).

The stomata-related parameters were analyzed by using software (version 1.8.0, ImageJ, available online at https://imagej.nih.gov/ij/download.html) according to previous definitions [35, 36]. In brief, the length and width of the guard cell, and the length and width of the pore, were measured based on the definition of Sack [35]. The guard cell area was presented as the length of guard cell multiplied by the width of the guard cell pair, while the pore area was calculated as the pore length multiplied by two times of the pore width [37]. The stomata density and number of stomata per area were calculated as the stomata number divided by the measured area, where the stomata number was counted. The pore area per leaf area was presented as the total pore area divided by the recorded area, where the stomata number was counted. The stomatal aperture was calculated as the pore width divided by the pore length [36].

### 2.3. Measurements of Soluble Sugar, Starch, Soluble Proteins, Total Phenols, and Flavonoids

The contents of soluble sugar and starch were measured by the anthrone colorimetric method as described by Xue et al. [38]. Soluble proteins were extracted by a sodium phosphate buffer and then measured colorimetrically by the Bradford method based on previous publication [39]. Total phenols and flavonoids were extracted with 80% methanol. The contents of total phenols and flavonoids were estimated by the previously described methods by Manivannan et al. [40].

### 2.4. Localization of *H*_2_*O*_2_ by DAB Staining and Antioxidant Enzyme Activity Assays

Excised leaves were immersed in a 0.1% 3,3′-diaminobenzidine (DAB) solution containing 0.05% Tween 20 and 0.0175% H_2_O_2_, followed by a vacuum infiltration for 15 min and an incubation in dark condition for 2 hours. Then leaves were washed carefully three times with distilled water and then immersed in absolute ethanol. Finally, leaves were boiled in hot water until all leaves become white. The activities of sodium dismutase (SOD), catalase (CAT), and guaiacol peroxidase (GPX) were determined according to the protocols described by Muneer et al. and Soundararajan et al. [34, 41], respectively.

### 2.5. Quantifications of Photosynthetic Components and Immunoblot Analysis

Chlorophyll content was determined by using a chlorophyll meter (SPAD-502 Plus, Konica Minolta Inc., Osaka, Japan). Chloroplast proteins were extracted based on the methods of Muneer [42, 43] and then separated by sodium dodecyl sulfate PAGE (SDS-PAGE). The intensity of protein bands was analyzed by using software (version 1.8.0, ImageJ, National Institutes of Health, Bethesda, MD, USA, https://imagej.nih.gov/ij/download.html) [39]. For western blots of D1 protein, the protein was extracted by a 100 mM Tris-HCl buffer (pH 7.8) with 1 mM EDTA-Na_2_, 2% PVP, 1% triton X-100, and 0.07% *β*-mercaptoethanol according to a previously described protocol [28, 41]. Extracted protein (25 *μ*g) was mixed well with a 240 mM Tris-HCl buffer (pH 6.8) containing 8% sodium dodecyl sulfate (SDS), 0.04% bromophenol blue, 40% glycerol, and 5%  *β*-mercaptoethanol and then separated by the SDS-PAGE. Finally, the expression of D_1_ protein (anti-PsbA) was analyzed by the immunoblotting described by Muneer [42]. The extracted proteins for each sample were loaded on the gel using an equal soluble protein basis. And the contents of proteins were shown as a percentage relative to HPS.

### 2.6. Statistical Analysis

The experimental assays were performed with three times individual biological repeats and data were presented as the mean ± standard error (SE) of the mean. Data were statistically analyzed by one-way analysis of variance (ANOVA), followed by Duncan's multiple range test at p < 0.05, using a statistical analysis software (V. 9.12, Statistical Analysis System, Cary, NC, USA).

## 3. Results

### 3.1. Spectral Characteristics of Different Supplementary Light Sources

Different artificial light sources provide various spectral characteristics which may differ from the sunlight. In this study, the five supplementary light sources used clearly displayed diverse spectral features ranging from 250 to 1,050 nm (Figures 1(a)–1(e) and Table 1). Irradiance of the HPS was mainly in green band (41.6%) followed by red (29.7%) and blue (only 4.8%) bands (Figures 1(a) and 1(f), and Table 1). Similarly, the MH also showed a high percentage of irradiance in green band (41.1%). However, the MH had a higher proportion of blue (30.9%) and less red (12.3%) irradiance than the HPS. Moreover, irradiance of the FR was mainly concentrated in the wavelength range from700 to 1,050 nm (FR and IR) followed by 600 to 700 nm (red). The distribution of irradiance of the LED-w exhibited a broader band from 400 to 700 nm and had a distinct peak (at 453 nm) in blue. In contrast to the LED-w, the LED-mix possessed a narrow spectrum with two peaks at 454 and 660 nm, and irradiance was mainly distributed in blue (44.4%) and red (43.1%) bands.

### 3.2. Growth, Development, and Morphology

**Figure 2:**
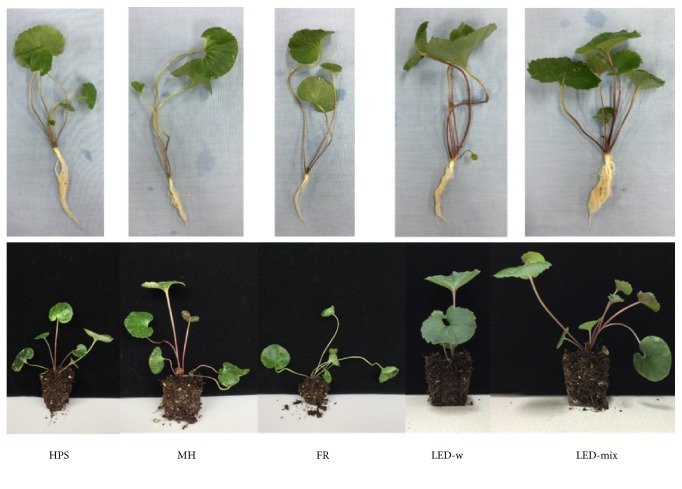
Morphological characteristics of* A. triphylla* seedlings grown with different supplementary light sources: HPS, high pressure sodium; MH, metal halide; FR, far-red; LED-w, white (red: green: blue = 2:4:3) light-emitting diodes; and LED-mix, mixed (red: green: blue = 4:1:4) light-emitting diodes.

In this study, growth and morphological characteristics of the seedlings were greatly influenced by supplementary light source (Figure 2 and Table 2). Stem diameter in the LED-mix was 3.8±0.2 mm, remarkably greater than that in the other treatments except the MH. Biomass, including total dry weight and shoot dry weight, showed a similar pattern that biomass in the LED-mix, LED-w, and MH was greater than that in the HPS and FR. More importantly, seedlings in the LED-mix presented strength to hold medium and to form a bigger root ball (Figure 2). Meanwhile, seedlings in the LED-mix had a significantly greater specific leaf weight (2.20±0.08 × 10^−2^ g·cm^−2^) than that in the HPS, MH, and FR (p < 0.05). Total seedling length showed no differences among treatments except the FR. The root to shoot ratio in the LED-mix was the greatest (0.42±0.05) while there were no differences in number of leaves among the treatments (p = 0.06 > 0.05).

### 3.3. Anatomical Feature of Stomata

**Figure 3:**
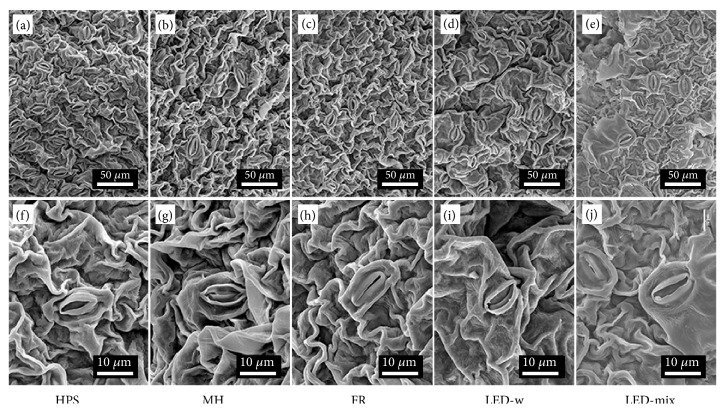
Stomatal development in* A. triphylla* leaves after four weeks of cultivation under different supplementary light sources: HPS, high pressure sodium ((a) and (f)); MH, metal halide ((b) and (g)); FR, far-red ((c) and (h)); LED-w, white (red: green: blue = 2:4:3) light-emitting diodes ((d) and (i)); and LED-mix, mixed (red: green: blue = 4:1:4) light-emitting diodes ((e) and (j)). Bar, 10 ((f)–(j)) or 50 ((a)–(e)) *μ*m.

The stomatal characteristics were influenced by supplementary light source (Figure 3 and Table 3). The highest stomata density was found in the LED-mix (664.1±33.4 mm^−2^) followed by the HPS and LED-w. Stomatal density in the FR and MH is considerably lower than the other treatments. Adversely, the aperture of stomata showed a contrary feature except in the FR. The smallest aperture was found in the LED-mix, only 0.15±0.03, while the MH had the greatest aperture (0.26±0.02) followed by the LED-w and HPS. In addition, the pore length and the guard cell area in the MH were remarkably greater than those in other treatments. More importantly, the pore area per leaf area in the LED-w, LED-mix, and HPS was markedly greater than in the MH and FR. However, there was no difference in the guard cell length, guard cell width, pore width, and pore area among the treatments.

### 3.4. Estimation of Photosynthetic Components

**Figure 4:**
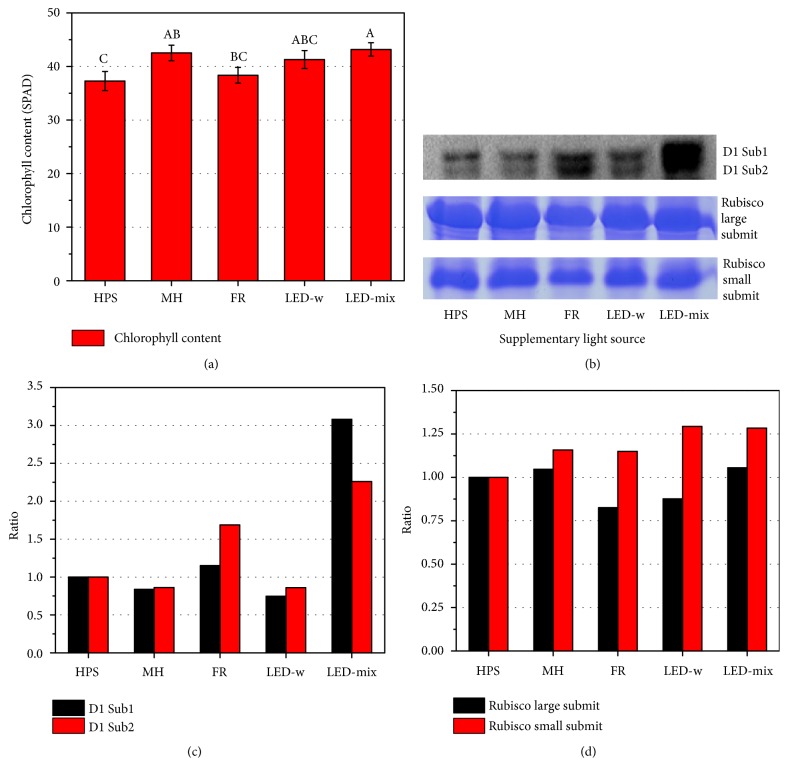
Effect of supplementary light source on chlorophyll content (a), expression (b), and relative contents of D1 protein (c) and Rubisco (d) in leaves of* A. triphylla* seedlings. D1 protein was analyzed by the Western blot/immunoblots analyses, while Rubisco was analyzed by sodium dodecyl sulfate polyacrylamide gel electrophoresis (SDS-PAGE). The extracted proteins for each sample were loaded on the gel using an equal soluble protein basis. The contents of proteins were shown as a percentage relative to HPS. HPS, high pressure sodium; MH, metal halide; FR, far-red; LED-w, white (red: green: blue = 2:4:3) light-emitting diodes; and LED-mix, mixed (red: green: blue = 4:1:4) light-emitting diodes.

The data showed that supplemental light source impacted the chlorophyll content in leaves (Figure 4(a)). The greatest chlorophyll content (43.18±1.25 SPAD) was obtained in the LED-mix followed by the order of MH, LED-mix, FR, and HPS (Figure 4(a)). The chloroplast protein profile showed about 30 apparent proteins, with the molecular mass ranging from 10 to 175 kDa (Figure S1). They were ordered from the largest to the smallest based on their molecular masses and presented by their intensities (Figures S1B-G). As compared with the HPS, expression of most proteins in the MH (90%) and LED-mix (80%) was maintained at a higher level. Meanwhile, there were only 10% proteins in the FR and 50% in the LED-w higher than in the HPS.

The Western blot analysis revealed that the expression of D1 protein was higher in the LED-mix followed by the FR and HPS and was relatively lower in the LED-w or MH (Figure 4(b)). In detail, the expression of Sub1 and Sub2 in the LED-mix was 3.08 and 2.26 times as high as that in the HPS (Figure 4(c)), respectively. In the LED-w, the expression of Sub1 and Sub2 showed the lowest values, which were 0.75 and 0.86 times, respectively, the amount in the HPS (Figure 4(c)). The SDS-PAGE analysis showed that the expression of Rubisco in LED-mix was improved and Rubisco large submit and small submit were 1.06 and 1.28 times as high as that in HPS, respectively.

### 3.5. Contents of Soluble Sugar, Starch, and Soluble Proteins

The supplemental light source had influence on primary metabolites. As shown in Table 4, soluble sugar content in the LED-mix was the greatest (2.31±0.18%), which was 1.34 and 1.19 folds greater than that in the FR and MH, respectively. Meanwhile, content of starch in the LED-mix (1.13±0.04%) was greater than that in other treatments. However, there were no obvious differences in content of soluble proteins.

### 3.6. Contents of Total Phenols and Flavonoids

**Figure 5:**
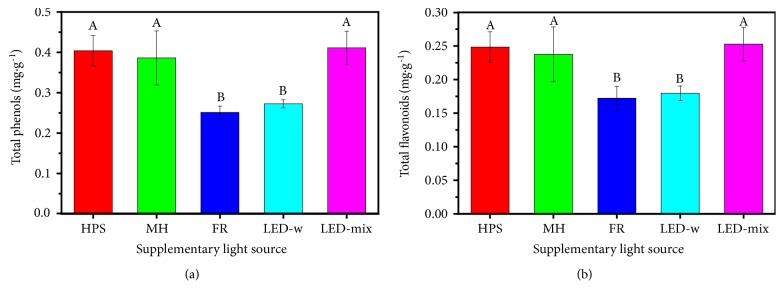
Contents of total phenols (a) and flavonoids (b) in* A. triphylla* leaves affected by supplementary light source: HPS, high pressure sodium; MH, metal halide; FR, far-red; LED-w, white (red: green: blue = 2:4:3) light-emitting diodes; and LED-mix, mixed (red: green: blue = 4:1:4) light-emitting diodes. Data are presented as the mean ± standard error of mean (n = 3). Different letters (A and B) indicate significant differences (p < 0.05) among treatments.

The supplementary light source not only affected the primary metabolism, but also influenced the secondary metabolism. In this study, the greatest content of total phenols (0.41±0.04 mg·g^−1^) was found in the LED-mix followed by the HPS and MH and was significantly greater than that in the LED-w and FR (Figure 5(a)) (p < 0.05). Similarly, total flavonoid content showed a similar tendency. Content of total flavonoid in the LED-mix was 0.25±0.03 mg·g^−1^, which was greater than that in the HPS, MH, LED-w, and FR (Figure 5(b)).

### 3.7. Localization of *H*_2_*O*_2_ by DAB Staining and Activities of Antioxidant Enzymes

**Figure 6:**
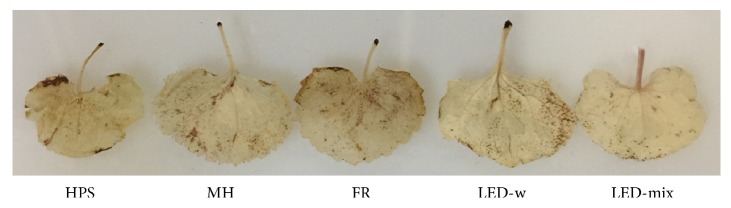
Histochemical localization of hydrogen peroxide by DAB staining in* A. triphylla* leaves grown under different supplementary light sources: HPS, high pressure sodium; MH, metal halide; FR, far-red; LED-w, white (red: green: blue = 2:4:3) light-emitting diodes; and LED-mix, mixed (red: green: blue = 4:1:4) light-emitting diodes.

In order to evaluate the level of abiotic stress under different supplementary light sources, the H_2_O_2_ in leaves were localized. As shown in Figure 6, the leaf in the FR showed a relatively deep brown appearance, followed by the HPS, implying a high level of H_2_O_2_ in leaf cells. Meanwhile, an alleviated symptom was found in the MH and LED-w treatments as compared with other treatments. However, the leaf grown in the LED-mix exhibited a light brown feature, suggesting less stress from the light spectrum and a better condition for growth.

**Figure 7:**
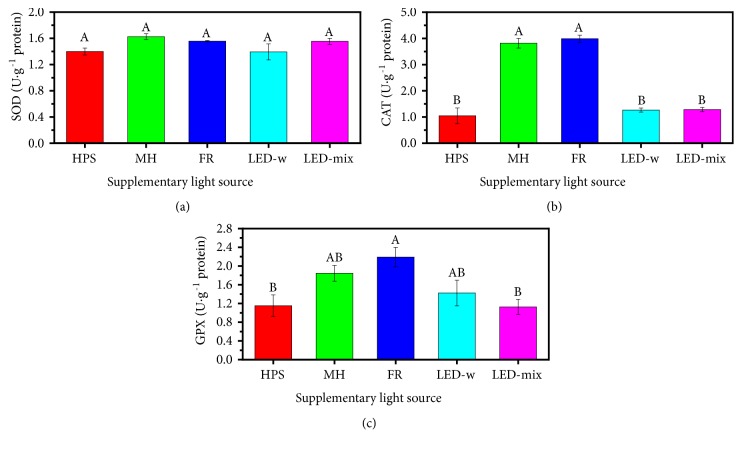
Activities of SOD (a), CAT (b), and GPX (c) in* A. triphylla *leaves grown under different supplementary light sources: HPS, high pressure sodium; MH, metal halide; FR, far-red; LED-w, white (red: green: blue = 2:4:3) light-emitting diodes; and LED-mix, mixed (red: green: blue = 4:1:4) light-emitting diodes. SOD, sodium dismutase; CAT, catalase; and GPX, guaiacol peroxidase. Data are presented as the mean ± standard error of mean (n = 3). Different letters (A and B) indicate significant differences (p < 0.05) among treatments.

In order to evaluate the scavenging activity of ROS, activities and expressions of antioxidant enzymes such as SOD, CAT, and GPX were measured as shown in Figure 7. The results showed no differences in the SOD activity although the value in the MH and FR treatments was slightly greater than in other treatments (Figure 7(a)). However, the MH and FR significantly promoted the activities of CAT (3.81±0.18 and 3.99±0.14 U·g^−1^ protein, respectively) as compared to the other three treatments (Figure 7(b)) (p < 0.05). Similarly, the highest activity of GPX was observed in the FR treatment (2.19±0.21 U·g^−1^ protein), followed by the MH and LED-w, whereas the LED-mix (1.13±0.16 U·g^−1^ protein) and HPS (1.15±0.23 U·g^−1^ protein) had remarkably lower values as compared with the FR (Figure 7(c)).

## 4. Discussion

Supplementary light source is of importance in indoor farming because different light lamps provide specific spectra and greatly influence the quality and quantity of crops grown under those lamps [44, 45]. Thus, choosing the optimal light source is crucial for maximizing crop productivity. [46]. Traditionally, the HPS and MH catch the growers' attention, since these two lamps provide broad ranges of light with lower prices (Figure 1). However, one main disadvantage is the low photoelectric conversion efficiency and higher electricity costs [19, 47]. The green light accounted for more than 40% of the HPS and MH (Table 1), which is far away from the optimum light quality for plant growth and development. Previous studies have established that red and blue light can be perceived by photoreceptors, such as phytochrome, cryptochrome, phototropins, and members of the Zeitlupe family, and be absorbed more efficiently by the plant for photosynthesis [11, 18, 48–51]. Therefore, in order to improve the electron transfer efficiency and to provide special bands for plant growth and development, the LED was introduced and employed in horticulture production [27, 52]. Our data showed that a total of red and blue light in the LED-mix took up 87.5%, which is higher than that in other treatments (Figure 1(f)) and may benefit photosynthesis in leaves and thus for growth and development of seedlings.

The supplementary light source had various effects on growth, development, and morphology of* A. triphylla* seedlings (Figure 2) [31]. The data showed that the LED-mix improved the quality of seedlings by increasing stem diameter, biomass, specific leaf weight, and root to shoot ratio (Table 2). The probable reason was that LED-mix could provide a higher percentage of red and blue light, increasing light use efficiency for photosynthesis, as compared with other supplementary light sources. Furthermore, high proportions of red and blue light also benefit the development of stomata and photosynthetic components (Figures 3 and 4), which improve photosynthesis and thus seedling quality. The results are in agreement with those in previous studies [10, 33, 53, 54]. However, some studies reported contrary results [24, 29, 55]. Bergstrand et al. [24] reported that tomato and rose crops were improved under the HPS in terms of biomass, plant height, and leaf area. Shao et al. [29] showed a low dry weight in* Gynura bicolor* under the LEDs as compared with the HPS and T5 fluorescent lamps. Alsanius et al. [55] found that all growth parameters were lower in sunflowers (*Helianthus annuus* L.) exposed to the LEDs than the HPS. Several factors could have contributed to these differences in results. The first reason probably is the difference in species, since different species have variation in photosensitivity [30, 31]. Secondly, temperatures used in their experiment (about 18 or 20°C in the daytime) are lower than those (31.6/26.3°C day/night) used in our study and the HPS can raise leaf temperature which may be beneficial for plant growth and development in relatively low temperatures [55–57]. As compared with the LED-w, a positive effect on stem diameter and root biomass was found in the LED-mix, because of an increased red and a decreased green light portion (Figure 1(f)). However, these results are consistent with others [58–60]. For example, Bian et al. [58] showed that RB LED (R:B = 4:1) was more effective than the white LED in improving lettuce growth. Zhang et al. [60] found that green light and yellow light inhibited the growth of lettuce. Nawaz et al. [59] found that red light enhanced, while green light suppressed, radicle growth in* Brassica rapa*.

Stomata are of importance in plant functioning, since they control gas exchange with the atmosphere and influence two basic physiological processes, photosynthesis and transpiration [61–63]. Previous studies found that supplementary light source affected formation, development, and functioning of stomata [17, 32, 64]. Stomatal density and aperture are two limiting factors in plant growth and are also two important indicators of plant adaptation and acclimation to environment [35, 65]. In our study, leaves grown in the LED-mix had greatest stomatal density, implying that the LED-mix improved the formation and development of stomata (Table 4). A high percentage of blue light in the LED-mix might have contributed to a high stomatal density, since blue light was reported to be the most beneficial on the aperture and number of stomata [66]. Zheng et al. [9] presented that, as compared with red light, blue light increased stomatal density and stomatal index and enhanced stomatal conductance in* Cordyline australis*,* Ficus benjamina*, and* Sinningia speciosa*. In addition, a low percentage of green light in the LED-mix might have a positive effect on stomatal density. Jensn et al. [17] reported that stomatal density increased with increasing ratio of blue light, while it decreased with green light in* Ocimum basilicum* L. Importantly, an increase in stomatal density can improve the gas exchange rate, leaf photosynthetic capacity, and photosynthetic rate [11, 36, 67, 68]. This partly explains enhancement of seedling quality of* A. triphylla* found in our study. On the other hand, stomatal aperture is a limiting factor in photosynthesis and plant growth [65]. However, our data showed a minimum value in the LED-mix (Table 4). Similarly, Zu et al. [69] found that supplemental UV-B radiation increased stomatal density, while decreasing stomata aperture in* Taxus chinensis* var. Mairei. Although stomatal aperture in the LED-mix was low in this study, the pore area per leaf area was maintained at a high level which ensured sufficient substrates and suitable gas exchange rate for photosynthesis (Table 4), indicating a fine regulation of gas exchange.

As an important environmental factor, light greatly influences photosynthesis and thus yield since plant growth and yield depend on photosynthesis [6, 7, 12]. Obviously, enhancement of photosynthetic efficiency is of vital importance to increase crop productivity to meet a rising demand of human [70]. The photosynthetic apparatuses, located in the thylakoid, include several integral membrane protein complexes, such as photosystem I (PSI), photosystem II (PSII), cytochrome b6/f, and ATP synthase [13, 71]. The PSII reaction center protein D1, encoded by the* psbA* gene, plays a key role in the initiation of photosynthesis and photosynthetic electron transport and thus greatly affects photosynthetic efficiency [13, 72, 73]. In our research, the greatest abundance of D1 protein (PsbA) was observed in the LED-mix, implying a higher level of photosynthetic efficiency as compared with others (Figure 4). Moreover, chlorophyll content in the LED-mix was also higher than that in the others, suggesting a stronger capacity for photosynthesis [31]. As a green pigment, chlorophyll is an essential compound of light-harvesting complex (LHC) in the PSI and PSII, which absorb photons and transfer light energy to the reaction center of photosystems [71, 74]. Hence, an increase in chlorophyll content in the LED-mix indicates enhancement of photosynthetic apparatus integrity and light-harvesting efficiency [8]. Efficient absorption and transfer of light energy by the chlorophyll increase the photosynthetic efficiency and then enhance photosynthesis. Thus, the chlorophyll content is closely related to the photosynthetic capacity [75]. Furthermore, as the first key enzyme in carbon fixation, Rubisco was improved in LED-mix, which obviously benefitted the photosynthesis process (Figure 4). Therefore, LED-mix enhanced the production of photosynthetic components such as chlorophyll, D1, and Rubisco, leading to an improved photosynthesis and thus a high quality of seedlings.

Supplementary light source affected accumulation of primary metabolites [20, 28]. Our data showed that the LED-mix enhanced contents of soluble sugar and starch (Table 3), which was in line with the accumulation of biomass (Table 2). The main reason is that the LED-mix could enhance the capacity of photosynthesis by increasing chlorophyll content and expressions of D1 and Rubisco (Figure 4), as compared with other treatments. As a photosynthetic product synthesized by photosynthetic process and a substrate consumed by the respiration, carbohydrate accumulation including soluble sugar and starch is important in plant growth, development, and morphology (Figure 2). Similar results have been found in other studies. For example, as compared with the plants grown in the HPS, plants grown in the LED exhibited 20% higher capacity of photosynthesis and higher levels of soluble carbohydrates in* Rosa x hybrida* leaves [31]. Moreover, Mao et al. [76] reported that a mixed LED (8R2B) had the highest levels of carbohydrates and lipids and largely promoted the growth of* Arthrospira platensis* as compared with the white LED. Similarly, combinations of R and B significantly stimulated carbohydrate accumulation in tomato (*Solanum lycopersicum*) [77],* Doritaenopsis* [78],* Ageratum houstonianum*,* Tagetes erecta*, and* Salvia splendens *[16], as compared with the white LED, monochromic LED, or fluorescent light.

The accumulation of secondary metabolites is also known to be regulated by supplementary light source [15, 79]. In our study, the highest levels of total phenols and flavonoids were found in the LED-mix (Figure 5). A number of studies showed a similar trend. For instance, Ouzounis [20] found that a 40% B+60% R LED increased contents of all phenolic acids and flavonoids in* Rosa hybrida*,* Chrysanthemum morifolium*, and* Campanula portenschlagiana* as compared with the white or monochromatic LED. It also worked in a similar way in* Brassica rapa* [59], amaranth (*Amaranthus* spp.) sprouts [80], and strawberry fruit [81]. The reason is that high blue light in LED-mix greatly contributes to the increase of the secondary metabolites such as phenols and flavonoids as compared with other treatments [26, 82, 83]. More studies suggested that blue light should be recommended as a supplementary light source for accumulation of phenols and flavonoids [84, 85]. Moreover, the promotion of primary metabolism partly promoted contents of phenols and flavonoids in the LED-mix treatment (Table 3), since the products of primary metabolism are utilized to synthesize a wide range of secondary metabolites through the shikimate pathway, mevalonic acid pathway, and methylerythritol 4-phosphate (MEP) pathway in higher plants [86–89]. Results from Zhao et al. [90] showed that enhanced accumulation of most secondary metabolites by elevated temperature is possibly due to increased contents of chlorophyll, sugar, and starch in* Robinia pseudoacacia* seedlings.

The relationship between reactive oxygen species (ROS) and abiotic stresses in the plant has been well documented [41, 71, 91]. In our research, the localization of H_2_O_2_ in leaves showed that oxidation stress increased in the FR and HPS, followed by the MH and LED-w, while it decreased in the LED-mix treatment (Figure 6). A high level of ROS is destructive for photosynthesis, because ROS reduces the abundance of D1 protein in leaves by suppressing biosynthesis and inducing degradation of D1 protein [72, 92]. This partly explains a high level of D1 protein in the LED-mix with a low level of H_2_O_2_ (Figures 4 and 6). Moreover, increased ROS also leads to oxidative damage to DNA structure and cell membrane, subsequently leading to a physiological disorder [41, 91, 93], although low levels of ROS play a key role in developmental processes of plants by acting as a signal molecule [93, 94]. In our study, DAB staining showed a low level of H_2_O_2_ in the LED-mix treatment, suggesting that the LED-mix provided a more suitable light spectrum (red and blue light) for growth of* A. triphylla *seedlings. In order to scavenge excessive ROS and to protect them against oxidative damage, plants have evolved efficient enzymatic and nonenzymatic antioxidant systems [40, 57, 91]. In the enzymatic antioxidant system, the SOD catalyzes superoxide radicals into H_2_O_2_, which is further degraded into water and oxygen by the CAT or GPX. In this research, it was found that the activities of antioxidant enzymes were associated with oxidative stresses. For example, CAT activity is lower in plants grown under LED-w and LED-mix (Figure 7), while H_2_O_2_ accumulation seems to be lower in those treatments (Figure 6). One possible reason might be that plants grown under LED-mix have higher contents of photosynthetic components and thus would be able to use more light energy to drive electron transport to generate ATP and NADPH without dissipation of excess energy [95], leading to lower contents of antioxidant systems. Increases in the activities of antioxidant enzymes under oxidative stresses have been observed in other plants such as* Dianthus caryophyllus* [34],* Trifolium repens* [96], and* Solanum lycopersicum* [97]. In the nonenzymatic antioxidants system, total phenols and flavonoids are crucial compounds for ROS homeostasis in plants, because of a special structure donating electrons or hydrogens [98, 99]. In our study, higher contents of total phenols and flavonoids in the LED-mix might partly contribute to detoxification of ROS and to maintain the level of H_2_O_2_ low, thereby protecting seedlings against oxidative damage [20, 29]. Therefore, the LED-mix provided a beneficial light spectrum for growth and development of* A. triphylla *seedlings with a ROS homeostasis maintained by enzymatic and nonenzymatic antioxidant systems.

## 5. Conclusions

Supplementary light source is a pivotal factor influencing the quality and yield of crops, thus greatly affecting the interest of growers in commercial cultivations. This study demonstrated the effects of different supplementary light sources on the growth, metabolism, and physiology of* A. triphylla* seedlings. Our data suggest that the LED-mix, with a higher ratio of red and blue light, has greatly improved the quality of* A. triphylla* seedlings with a high biomass, compact stem, and well-developed roots. Moreover, the LED-mix significantly promoted the accumulation of primary and secondary metabolites, such as soluble sugar, starch, chlorophyll, total phenols, and flavonoids. Furthermore, results of an SEM analysis implied that the LED-mix has increased stomatal density and maintained a higher level of pore area per leaf area. Meanwhile the Western blot analysis suggested that the expression of photosynthetic protein, D1, was also promoted in the LED-mix. Additionally, the LED-mix mitigated the oxidative damage by maintaining a redox homeostasis regulated by enzymatic and nonenzymatic antioxidant systems. Collectively, the LED-mix was an alternative supplementary light source for the production of high quality* A. triphylla* seedlings.

## Figures and Tables

**Table 1:** The irradiance distributions of the different supplementary light sources used in this study.

Treatment	Irradiance (*μ*W·cm^−2^)					
	UV	Blue	Green	Red	FR	IR
	(250-400 nm)	(401-500 nm)	(501-600 nm)	(601-700 nm)	(701-800 nm)	(801-1050 nm)

HPS	16.7	145.0	1,258.5	900.4	151.2	557.0
MH	244.4	925.5	1,231.5	369.2	83.5	138.6
FR	2.1	0.7	1.5	155.0	563.3	2,245.6
LED-w	15.8	1,053.0	1,279.3	599.3	39.2	48.2
LED-mix	10.4	1,309.5	341.0	1,273.4	11.1	6.9

HPS: high pressure sodium; MH: metal halide; FR: far-red; LED-w: white LED (red: green: blue = 2:4:3); LED-mix: mixed (red: green: blue = 4:1:4) LED; UV: ultraviolet; and IR: infrared radiation.

**Table 2:** Growth characteristics of *A. triphylla *seedling grown under different supplementary light sources.

Treatment	Stem diameter (mm)	Total length (mm)	Total DW (g)	Shoot DW (g)	Root DW (g)	SLW b (10^−2^ g·cm^−2^)	No. of leaves	Root to shoot ratio

HPS	3.0 ± 0.2 b	207.9 ± 5.7 a	0.19 ± 0.02 b	0.15 ± 0.01 b	0.04 ± 0.01 c	1.95 ± 0.06 b	4.8 ± 0.4 a	0.30 ± 0.04 b
MH	3.4 ± 0.2 ab	211.3 ± 7.7 a	0.31 ± 0.03 a	0.24 ± 0.02 a	0.07 ± 0.01 ab	1.94 ± 0.09 b	6.6 ± 0.6 a	0.30 ± 0.04 b
FR	2.2 ± 0.1 c	172.9 ± 8.5 b	0.11 ± 0.01 c	0.09 ± 0.01 c	0.02 ± 0.00 d	1.53 ± 0.09 c	4.7 ± 0.5 a	0.21 ± 0.03 b
LED-w	3.0 ± 0.1 b	216.5 ± 10.6 a	0.28 ± 0.03 a	0.24 ± 0.02 a	0.05 ± 0.01 bc	2.21 ± 0.09 a	5.6 ± 0.7 a	0.22 ± 0.02 b
LED-mix	3.8 ± 0.2 a	199.8 ± 5.3 a	0.30 ± 0.03 a	0.22 ± 0.02 a	0.08 ± 0.01 a	2.20 ± 0.08 a	6.0 ± 0.4 a	0.42 ± 0.05 a

DW: dry weight; SLW: specific leaf weight; HPS: high pressure sodium; MH: metal halide; FR: far-red; LED-w: white (red: green: blue = 2:4:3) LED; and LED-mix: mixed (red: green: blue =4: 1: 4) LED. Data are presented as the mean ± standard error of mean (n = 10). Different letters (a, b, and c) indicate significant differences (p < 0.05) among treatments.

**Table 3:** Stomata characteristics in *A. triphylla* leaves after four weeks of cultivation under different supplementary light sources.

Treatment	SD (mm^−2^)	GCL (*μ*m)	GCW (*μ*m)	PL (*μ*m)	PW (*μ*m)	GCA (*μ*m^2^)	PA (*μ*m^2^)	PAPLA (1000 *μ*m^2^·mm^−2^)	Aperture

HPS	433.9 ± 21.2 b	11.9 ± 1.2 a	2.6 ± 0.6 a	15.5 ± 0.4 b	3.8 ± 0.2 a	71.5 ± 1.3 c	28.6 ± 8.7 a	12.8 ± 4.5 a	0.22 ± 0.03 ab
MH	121.1 ± 31.7 c	11.5 ± 3.2 a	2.9 ± 0.6 a	21.0 ± 0.3 a	4.1 ± 0.1 a	116.9 ± 8.9 a	29.8 ± 12.1 a	4.2 ± 2.0 b	0.26 ± 0.02 a
FR	98.8 ± 20.5 c	12.5 ± 1.0 a	2.3 ± 0.2 a	17.3 ± 1.5 b	3.7 ± 0.2 a	80.1 ± 4.6 bc	25.6 ± 2.0 a	2.6 ± 0.7 b	0.19 ± 0.03 ab
LED-w	414.2 ± 10.1 b	13.5 ± 0.2 a	3.4 ± 0.4 a	17.5 ± 0.3 b	4.0 ± 0.3 a	80.7 ± 4.1 bc	38.4 ± 5.9 a	15.8 ± 2.9 a	0.25 ± 0.03 a
LED-mix	664.1 ± 33.4 a	13.0 ± 1.1 a	1.9 ± 0.2 a	17.3 ± 0.5 b	4.0 ± 0.1 a	98.6 ± 3.5 ab	20.9 ± 2.2 a	13.8 ± 1.0 a	0.15 ± 0.03 b

SD: stomata density; GCL: guard cell length; GCW: guard cell width; PL: pore length; PW: pore width; GCA: guard cell area; PA: pore area; and PAPLA: pore area per leaf area (1000 *μ*m^2^·mm^−2^). HPS: high pressure sodium; MH: metal halide; FR: far-red; LED-w: white (red: green: blue = 2:4:3) light-emitting diodes; and LED-mix: mixed (red: green: blue = 4:1:4) light-emitting diodes. Different letters (a, b, and c) indicate significant differences (p < 0.05) among treatments.

**Table 4:** Contents of soluble sugar, starch, and soluble protein in *A. triphylla* leaves as affected by supplementary light source.

Treatment	Soluble sugar (% of FW)	Starch (% of FW)	Soluble protein (% of FW)

HPS	2.16 ± 0.05 ab	1.01 ± 0.04 b	0.87 ± 0.07 a
MH	1.94 ± 0.04 ab	1.04 ± 0.02 ab	0.80 ± 0.06 a
FR	1.73 ± 0.20 b	1.00 ± 0.01 b	0.85 ± 0.05 a
LED-w	2.27 ± 0.09 a	1.10 ± 0.02 ab	0.84 ± 0.04 a
LED-mix	2.31 ± 0.18 a	1.13 ± 0.04 a	0.83 ± 0.02 a

FW: fresh weight; HPS: high pressure sodium; MH: metal halide; FR: far-red; LED-w: white (red: green: blue = 2:4:3) LED; and LED-mix: mixed (red: green: blue = 4:1:4) LED. Data are presented as the mean ± standard error of mean (n = 9). Different letters (a and b) indicate significant differences (p < 0.05) among treatments.

## Data Availability

The data used to support the findings of this study are available from the corresponding author upon request.

## Supplementary Materials

Supplementary Materials Figure S1: Expression of chloroplast proteins (A) and relative contents (B-G) in leaves of* A. triphylla* seedlings grown under different supplementary light sources analyzed by sodium dodecyl sulfate polyacrylamide gel electrophoresis (SDS-PAGE). HPS, high pressure sodium; MH, metal halide; FR, far-red; LED-w, white (red: green: blue = 2:4:3) light-emitting diodes; and LED-mix, mixed (red: green: blue = 4:1:4) light-emitting diodes. The extracted proteins for each sample were loaded on the gel using an equal soluble protein basis. The contents of proteins were shown as a percentage relative to HPS. Band 10, Rubisco large subunit; Band 29, Rubisco small subunit.
