# Supplementary figures and images for: Supplementary Light Source Affects Growth, Metabolism, and Physiology of Adenophora triphylla (Thunb.) A.DC. Seedlings
Source: Biomed Res Int. 2019 May 7;2019:6283989. doi: 10.1155/2019/6283989 (PMC6530224; doi:10.1155/2019/6283989)

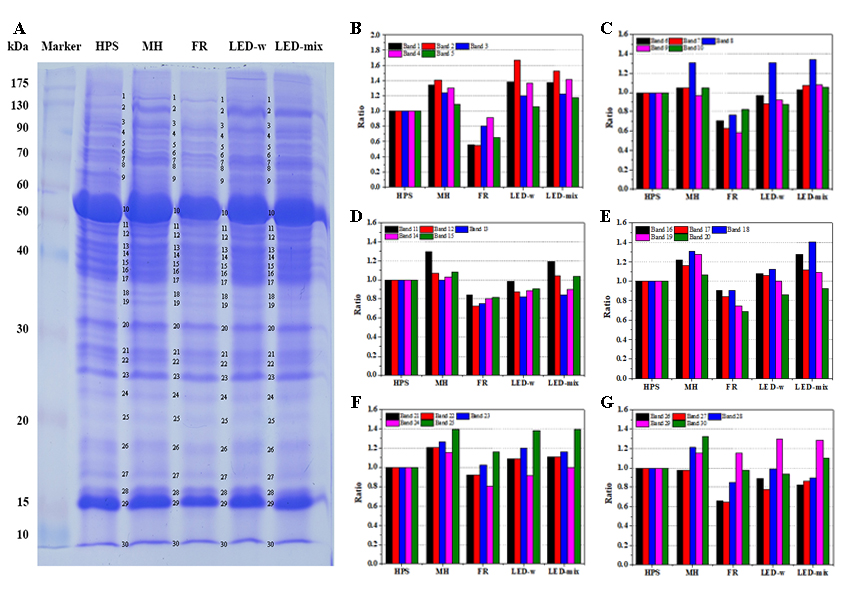

Supplement: Supplementary Materials — Figure S1: Expression of chloroplast proteins (A) and relative contents (B-G) in leaves of A. triphylla seedlings grown under different supplementary light sources analyzed by sodium dodecyl sulfate polyacrylamide gel electrophoresis (SDS-PAGE). HPS, high pressure sodium; MH, metal halide; FR, far-red; LED-w, white (red: green: blue = 2:4:3) light-emitting diodes; and LED-mix, mixed (red: green: blue = 4:1:4) light-emitting diodes. The extracted proteins for each sample were loaded on the gel using an equal soluble protein basis. The contents of proteins were shown as a percentage relative to HPS. Band 10, Rubisco large subunit; Band 29, Rubisco small subunit. [file 6283989.f1.jpg]
